# Algal Phlorotannins as Novel Antibacterial Agents with Reference to the Antioxidant Modulation: Current Advances and Future Directions

**DOI:** 10.3390/md20060403

**Published:** 2022-06-18

**Authors:** Biswajita Pradhan, Rabindra Nayak, Prajna Paramita Bhuyan, Srimanta Patra, Chhandashree Behera, Sthitaprajna Sahoo, Jang-Seu Ki, Alessandra Quarta, Andrea Ragusa, Mrutyunjay Jena

**Affiliations:** 1 Algal Biotechnology and Molecular Systematic Laboratory, Post Graduate Department of Botany, Berhampur University, Bhanja Bihar, Berhampur 760007, Odisha, India; pradhan.biswajita2014@gmail.com (B.P.); rabindran335@gmail.com (R.N.); chhandashreebehera@gmail.com (C.B.); 2 Department of Biotechnology, Sangmyung University, Seoul 03016, Korea; kijs@smu.ac.kr; 3 Department of Botany, Maharaja Sriram Chandra Bhanja Deo University, Baripada 757003, Odisha, India; prajnabhuyan2017@gmail.com; 4 Cancer and Cell Death Laboratory, Department of Life Science, National Institute of Technology Rourkela, Rourkela 769008, Odisha, India; 518LS2007@nitrkl.ac.in; 5 Department of Botany, Berhampur University, Berhampur 760007, Odisha, India; tibunsahoo@gmail.com; 6 CNR-Nanotec, Institute of Nanotechnology, Via Monteroni, 73100 Lecce, Italy; alessandra.quarta@nanotec.cnr.it; 7 Department of Biological and Environmental Sciences and Technologies, Campus Ecotekne, University of Salento, Via Monteroni, 73100 Lecce, Italy

**Keywords:** brown algae, marine algae, antibacterial activity, polyphenols, phlorotannin, antioxidant, antibiotic

## Abstract

The increasing drug resistance of infectious microorganisms is considered a primary concern of global health care. The screening and identification of natural compounds with antibacterial properties have gained immense popularity in recent times. It has previously been shown that several bioactive compounds derived from marine algae exhibit antibacterial activity. Similarly, polyphenolic compounds are generally known to possess promising antibacterial capacity, among other capacities. Phlorotannins (PTs), an important group of algae-derived polyphenolic compounds, have been considered potent antibacterial agents both as single drug entities and in combination with commercially available antibacterial drugs. In this context, this article reviews the antibacterial properties of polyphenols in brown algae, with particular reference to PTs. Cell death through various molecular modes of action and the specific inhibition of biofilm formation by PTs were the key discussion of this review. The synergy between drugs was also discussed in light of the potential use of PTs as adjuvants in the pharmacological antibacterial treatment.

## 1. Introduction

Synthetic antibiotics are frequently used to treat microbial diseases in humans but they often exert several side effects, such as renal dysfunction and cardiovascular diseases [1,2]. Therefore, the search for natural compounds in preventing various diseases is very promising [3,4,5,6,7,8,9,10,11,12]. The treatment of infectious bacterial diseases is even more critical if the patient suffers from concomitant pathologies, such as tuberculosis, pneumonia, salmonellosis, and gonorrhea, often leading to the patient’s death [13]. In such a hostile situation, the development of novel bioactive antimicrobial drugs with limited cytotoxicity, improved pharmacological efficacy, and immune to drug resistance is highly desired. Several studies already reported that marine algae can inhibit microbial growth [14,15,16,17]; therefore, they could act as natural antibiotics against human diseases.

The marine environment, as well as freshwater, is rich in both micro- and macro-algal biodiversity, which contains a variety of bioactive phyco-chemicals that could be utilized as anticancer, antioxidant, anti-inflammatory, antiviral, and antibacterial agents [18,19,20,21,22,23,24]. These naturally occurring compounds include proteins, vitamins, omega-3 fatty acids, and other antioxidant compounds, such as carotenoids, phenolic, polyphenolic, and flavonoid molecules [25,26]. Macro-algae are also potential candidates for producing a wide range of bioactive substances under various stress circumstances, with physiological and biochemical pathways that might be altered to preserve cellular homeostasis [27]. Reactive oxygen species (ROS) and other radical species produced by metabolism have a negative impact on organisms [28]. Several studies have reported on the impact of macroalgal bioactive substances on a variety of oxidative stress-related illnesses [29,30,31,32]. Numerous studies have linked oxidative stress to cancer, premature ageing, Alzheimer’s and Parkinson’s disease, as well as cardiovascular problems [33,34]. ROS play a critical role in signaling, inflammation-related bacterial diseases. As electron or hydrogen donors, antioxidants work as free radical scavengers by inhibiting oxyradical production through their conversion into stable compounds [35]. Polyphenols in natural compounds act as antioxidants and play a significant role in preventing or reducing the oxidation of biomolecules [36,37].

Previous studies have shown that brown seaweeds contain antimicrobial bioactive chemicals [38]. In addition, phlorotannins (PTs) also displayed promising antimicrobial properties [39]. According to literature [34,40,41], brown algae are a rich source of PTs with a wide range of biological activities, such as antibacterial, antifungal, antidiabetic, anticancer, and anti-inflammatory. The antibacterial capacity of PTs is largely influenced by the method of extraction, which determines the chemical structure of the phenolic compounds extracted [42]. In order to shed some light on these aspects, this article reviews the extraction methods employed to obtain PTs and their antibacterial activity, with particular reference to their mechanism of action and new exploitation approaches, such as their combined use with commercial antibiotics and also their anti-biofilm efficacy.

## 2. Structural Diversity of Phlorotannins

Marine algae are a very rich source of secondary metabolites with a variety of chemical structures and, correspondingly, a wide range of biological properties [11,43,44,45]. Marine brown seaweeds contain, among others, phloroglucinol and its polymers, such as PTs [46,47]. Nevertheless, red algae also contain 1.8–3.2% of PTs [48].

PTs are a type of tannin primarily found in brown algae, such as kelps, rockweeds, and *sargassacean* species, but also in small amounts in red algae. Phlorotannins are a special group of hydrophilic phenolic compounds that display strong binding efficacy to polysaccharides, proteins, biopolymers, and other chelate divalent metals. They exhibit a huge variety of chemical structures and, consequently, polymeric properties [49,50,51]. They resemble tannins from terrestrial plants and are thought to play a role in cell wall formation. Phlorotannins are made by polymerizing phloroglucinol (1,3,5-trihydroxybenzene) monomer units in a range of combinations, similarly to the well-studied biosynthesis of terrestrial plant tannins from alcoholic monomers [52,53,54]. However, the biochemical mechanism for phlorotannin biosynthesis is poorly understood, with several hypotheses ranging from acetate and malonate unit condensation to the shikimate or phenylpropanoid pathways. PTs can also be found in a sulfated or halogenated state and their biosynthesis is carried out in the Golgi apparatus of the cell via an acetate-malonate pathway [55].

**Figure 1:**
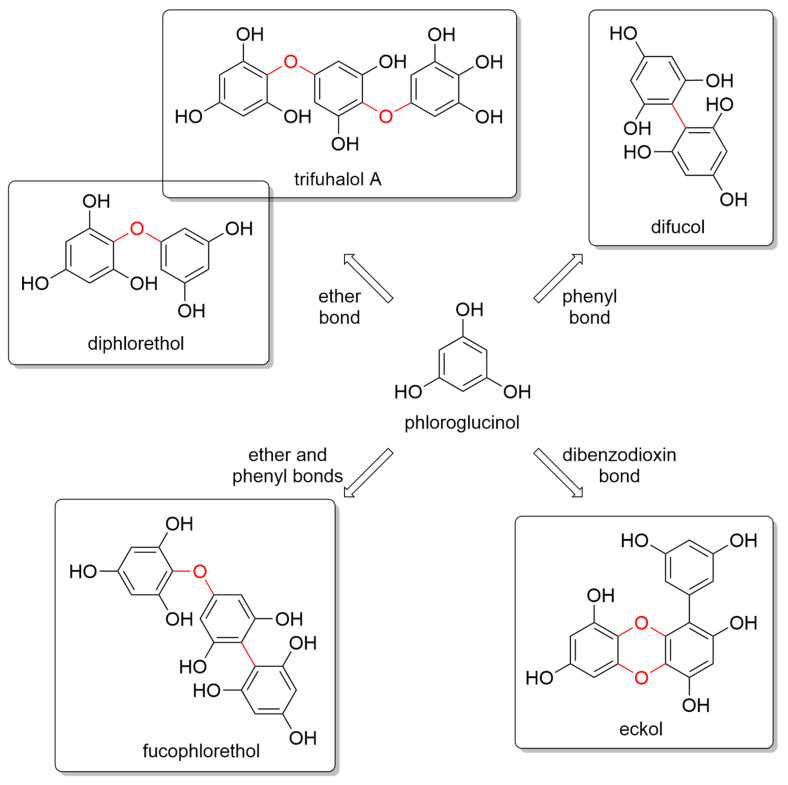
Monomeric phloroglucinol unit (**center**) and some representative components of the major four classes of phlorotannins according to their linkages, i.e., fuhalols and phlorethols (**top left**), fucols (**top right**), fucophlorethols (**bottom left**), and phloreckols (**bottom right**).

Glombitza was the first to develop a nomenclature scheme for marine phlorotannins according to the type of linkages and arrangement of the phloroglucinol monomers, the presence of an additional hydroxyl group for fuhalols, the existence of carmalols, and the potential presence of halogens, or sulfate groups [54,56]. PTs are generally divided into four main subclasses, i.e., phlorethols and fuhalols when joined through ether bonds, fucols when joined by phenyl bonds, fucophlorethols when they have both ether and phenyl bonds, and phloreckols when they contain dibenzodioxin bonds (Figure 1).

PTs are accumulated more in *Fucus* algae and constitute about 3–12% of its dry weight. In some algal species, this percentage reaches up to 20% of the algal dry weight [57,58,59]. The PTs have a broad range of molecular weight from 126 Da to 650 kDa depending on species, size, geographic region, tissue type, age, water salinity, season, nutrients, water temperature, light intensity, and extraction method. However, the most common molecular weight of PTs ranges from 10 to 100 kDa [60,61,62]. PTs are comprised of 1–15% of the thallus dry mass of algae [63].

## 3. Extraction Procedure of Polyphenols from Marine Algae

The extraction of polyphenols from seaweed is generally carried out by using polar organic solvents, such as ethanol, methanol, and acetone [39,48,64,65,66]. The most common solvents used for the extraction of PTs are aqueous solutions of acetone or ethanol [63,67]. Brown algae produce a wide range of polymers, but their physiological activity is unknown. To extract PTs, the optimal temperature must not exceed 52 °C as higher temperatures can lead to their degradation [48]. Because of the low selectivity of the target component, of long extraction times, and the necessity to purify further the extract, solid-liquid extraction procedures are commonly utilized for isolating PTs from algae [64].

Polyphenols from *Eisenia bicyclis*, as well as other forms of brown and red algae, were extracted with both distilled water and a mixture of methanol, water, and acetic acid (30:69:1 *v*/*v*/*v*) to obtain a significant quantity of polyphenols (about 193 mg/g gallic acid equivalents, GAE). Extraction by 80% methanol yielded the highest amount of polyphenols (about 15 mg/g GAE) from *Laminaria japonica*, whereas extraction with 100% methanol gave the highest yield of polyphenols (over 8 mg/g GAE) from *Undaria pinnatifida* [48]. The extraction of polyphenols from *Fucus evanescens* was higher when an aqueous solution of ethanol was employed as a solvent, while distilled water was utilized for the extraction of polyphenols from *S. japonica* and *Anfeltia tobuchiensis* [68]. Many previous works claim that extraction with methanol exhibited the highest yield of PTs [65,66]. Ethyl acetate also extracted high amount of PTs from *Sargassum fusiforme* (almost 90 mg phloroglucinol equivalents/100 mg of extract) [69]. Nevertheless, acetone also allowed for excellent extraction of PTs.

With traditional solvent-based methods of extraction, high-molecular weight PTs associated with the cell wall are not isolated [65,68]. Conversely, effective techniques for extracting polyphenols involve ultrasonication, enzymatic extraction, microwave, liquid extraction under pressure, and supercritical fluid extraction [64,66,68,70]. Among them, enzymatic extraction is very effective allowing algal cell wall destruction and high PTs recovery (21–38%) compared to solid-liquid extraction (3–15%) [66]. Mass transfer is stimulated during ultrasonic extraction by breaking plant cell walls, which enhances the release of high molecular weight PTs [64]. Microwave extraction has the benefit of yielding high amounts of polyphenols from plants while lowering extraction time and solvent use [64,66,68]. The time required to recover polyphenols is also greatly reduced when using high-pressure liquid extraction [39,48,64]. A considerable number of extraction methods have been employed for the extraction of PTs from algae, usually followed by chromatographic techniques for their purification [66]. To identify, quantify, and perform a structural analysis of PTs, nuclear magnetic resonance (NMR) spectroscopy and chromatography-mass spectrometry are generally used [70]. The existence of polysaccharide complexes as the principal component of the algal cell wall represents a substantial obstacle in polyphenols extraction, as PTs are included in the cell wall and are covalently bound to polysaccharides and proteins [71]. Nevertheless, modern chromatographic methods currently represent the state-of-the-art method for purifying and identifying PTs.

## 4. Antioxidant Properties of Algal Phlorotannins

PTs are biologically active compounds with anti-inflammatory, anti-allergic, antiviral, antitumor, antioxidant, antidiabetic, and radioprotective effects [72,73,74,75]. PTs from algal sources act as electron traps for free radicals [76], and display robust antioxidant activity thanks to the numerous hydroxyl groups, thereby being toxic to bacteria under aerobic conditions [77]. Ethanol extracts of algae from the genera *Agarum, Arthrothamnus, Fucus, Stephanocystis,* and *Thalassiophyllum,* showed effective antioxidant properties. PTs from *F. evanescens, Thalassiophyllum clathrus*, and *Stephanocystis crassipes* also exhibited significant antioxidant activity [64,68]. In addition, PTs extracted from the brown alga *Eisenia bicyclis* displayed 10 times higher antioxidant activity over ascorbic acid. The antioxidant activity of PTs depends largely on the molecular weight of the compounds [51,78,79].

Algal phenolic, polyphenolic, and flavonoid molecules stimulate a wide range of biological functions. Numerous biochemical assays have been exploited to assess the ability of PTs to scavenge free radicals, such as the 2,2-diphenyl-1-picryl-hydrazyl-hydrate (DPPH) free radical scavenging activity. When compared to ascorbic acid and α-tocopherol, PTs from brown algae *E. cava, E. kurome*, and *E. bicyclics* displayed considerable radical scavenging ability against the superoxide anion (Inhibitory Concentration–IC_50_–6.5–8.4 µM) and DPPH (IC_50_ 12–26 µM) [79]. The antioxidant activity of diphlorethohydroxycarmalol from brown algae *Ishige okamurae* was determined using the DPPH assay and the IC_50_ value was found to be between 3.41 and 4.92 mM [80]. The IC_50_ of PT fractions from *Sargassum ringgoldianum* against superoxide anion radicals was evaluated to be 1.0 mg/mL, which was about five times stronger than catechin [81]. 974-A, 974-B, phlorofucofuroeckol-A, and dieckol had significantly lower IC_50_ values than phlorofucofuroeckol-B, phloroglucinol, α-tocopherol, and ascorbic acid [82].

To date, natural antioxidants are considered harmless for human beings. In this regard, PTs have the ability to scavenge ROS such as peroxyl, hydroxyl, and superoxide radicals [83]. The DPPH free radical scavenging of *Sargassum aquifolium* displayed a maximum of almost 7 mg phlorotannin per g of dry weight extract compared to the approximately 6 mg/g obtained with ascorbic acid [84]. Phenolic compounds and PTs extracted from brown seaweed, such as *Trifucodiphlorethol, Trifucotriphlorethol,* and *Tucotriphlorethol*, were shown to have IC_50_ values ranging between 10 and 14 mg/mL [85]. PTs isolated from *E. cava* also showed promising antioxidant capacity [86]. The information presented here could serve to better understand the biological properties of *E. cava*, other marine brown seaweeds, and their derivatives, as well as their potential use as functional ingredients in industrial applications.

## 5. Mechanisms of Action of Phlorotannins Antibacterial Activity

**Figure 2:**
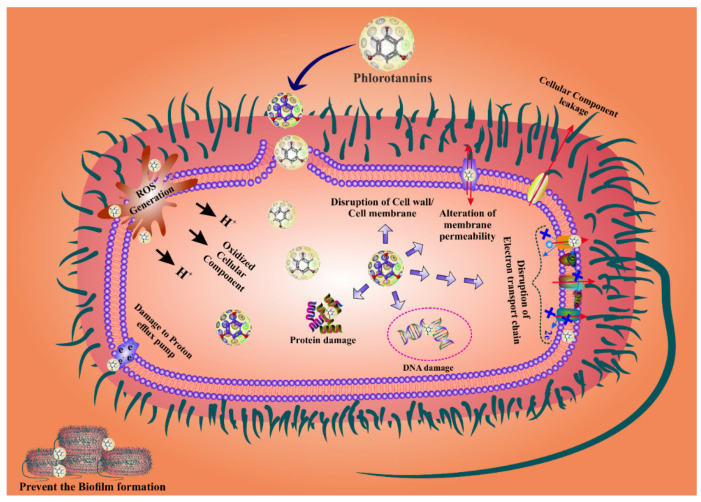
Phlorotannins display promising antibacterial activity by modulating cell death because of excessive ROS production. Moreover, PTs also prevent biofilm formation.

PTs are the most effective agents for fighting bacterial biofilms because they penetrate the bacterial cell wall by changing the shape of the cell membrane and causing cell death [87,88]. Bacterial cell wall permeability is damaged by PTs, which cause proton leakage in the cell membrane, thus structural changes in the nuclear membrane leading to bacterial cell death [53,89,90]. In addition, PTs have the ability to eradicate bacteria by inhibiting their reproduction. The antibacterial activity of PTs has been attributed to their capacity for blocking oxidative phosphorylation, as well as to their ability to attach to bacterial proteins and enzymes, causing cell lysis. The phenolic aromatic rings and the -OH groups of phloroglucinol bind to the -NH groups of bacterial proteins, leading to inhibition [91,92]. The presence of additional groups, such as the two acetyl residues in 2,4-diacetylphloroglucinol (DAPG) or the 1-methylvinyl residue at the C-3 of ialibinones, can improve the bacteriolytic activity of phloroglucinol compounds [93,94]. Several investigations found that PTs play an important role in suppressing bacterial reproduction [90]. In addition, they bind to bacterial RNA and DNA, again inhibiting bacterial replication [57]. PTs are most effective against gram-negative bacteria as they can bind to the thick coating of the peptidoglycan and the lipopolysaccharides present in these bacteria [67]. When PTs bind to the cell wall of gram-negative bacteria, their permeability changes [87,95,96]. Moreover, PTs can modify the bacterial phosphotyrosine, thus inactivating the protein and the DNA replication, finally leading to bacterial growth inhibition [97]. Some studies reported that PTs harm bacteria’s cell walls by forming a pit outside the cell, thus interfering with bacterial functions, including permeability and respiration, reducing the cell’s reproductive capability, and eventually leading to cell death [87,95,96]. PTs might downregulate the activity of antioxidant enzymes such as SOD, CAT, and GSH, disturbing the redox-homeostasis and subsequently inducing ROS-mediated cell death. However, the exact mechanism is still poorly understood. Hence, research studies should also focus in this direction in order to draw a comprehensive conclusion. The overall mechanism of the ROS-mediated bacterial cell death by PTs is displayed in Figure 2.

## 6. In Vitro Antibacterial Activity of Phlorotannins

Phlorotannins, and phenolic compounds in marine algae in general, have been shown to possess, among others, antibacterial activity, as summarized in Table 1.

PTs extracted from brown algae displayed vigorous antimicrobial activity with reference to phloroglucinol, eckol, and dieckol [55,107]. PT extracts inhibited both gram-positive and gram-negative bacteria [67]. Multiple investigations found that PTs derived from brown seaweeds have stronger antibacterial activity than traditional antibiotics against *Klebsiella*, *Bacillus cereus*, and *Pseudomonas aeruginosa* [108,109]. Ethyl acetate extracts from brown algae, such as *Ecklonia stolonifera* and *Ecklonia cava*, were shown to have antibacterial efficacy against methicillin-resistant *Staphylococcus aureus* (MRSA) [70]. Phlorofucofuroeckol-A from *E. bicyclics* also inhibited the growth of MRSA [38,98,99]. In addition, low molecular weight PTs isolated from *Sargassum thunbergii* displayed antibacterial activity against *Vibrio parahaemolyticus* by damaging the cell membrane and the cell wall, thus facilitating cytoplasm leakage and membrane permeability [100].

With a minimum inhibitory concentration (MIC) of 32 µg/mL, a phlorofucofuroeckol derivative displayed the most effective antibacterial action. In addition, it dramatically reduced resistance of *Propionibacterium* to erythromycin and lincomycin [101]. Moreover, phlorofucofuroeckol from *Eisenia bicyclis* displayed similar results against MRSA [102]. In resistant *Staphylococcus aureus* cells, phlorofucofuroeckol inhibited the expression of *mecI, mecR1,* and *mecA* genes and regulated the expression of methicillin resistance in bacteria by suppressing penicillin-binding protein 2a production [38,102]. Despite these promising preliminary results, substantial preclinical and clinical trials are necessary for assessing the therapeutic efficacy of the extracts in vivo.

## 7. Combination Therapy of PTs and Antibiotics: The Emerging Era of Drug Discovery

Drug synergism has been claimed as a feasible approach for improving the therapeutic efficacy of conventional drugs. Antimicrobial drug resistance has become one of the most severe public health issues in recent years [110]. In this regard, the rise of multidrug-resistant *S. aureus* pathogen strains poses a significant threat to the human society. In addition, *S. aureus* biofilms exert a serious risk of infecting patients in hospitals, as well as of contaminating the environment and the food. Antibiotics generally prevent the biofilm formation and increase the immunological response of the host [111,112]. Owing to drug-resistance, the discovery of natural products with potent antibacterial activity, either alone or in combination with conventional antibiotics, has become a high-priority research area in the current years [113,114]. In this context, seaweed is a promising source of polyphenols and PTs with antibacterial and antibiofilm potentials, showing immense potential as drug candidates with limited cytotoxicity [115,116]. Some antibiotics have shown synergistic effects with phenolic substances [117]. Various combinations of antibiotics and polyphenols can improve the efficacy of an antibiotic against a bacterial target [118]. In addition, the synergistic effect allows the reduction of the antibiotic’s working dose and as such its toxicity [119,120]. Using PTs in combination with antibiotics may be a viable approach for improving or restoring antibiotic efficacy in infections caused by multi-resistant bacteria, such as MRSA [95].

The permeability and integrity of the bacterial cell membrane and cell wall are altered by algal PTs, thus facilitating antibiotic entry into the cytoplasm [121,122]. Antibiotics can also stop bacteria from replicating, transcribing, and translating their DNA [122]. Therefore, the use of PTs in combination with antibacterial medicines can represent a promising multitarget strategy. Purified dieckol from the alga *E. stolonifera* showed a substantial synergistic effect with commercial β-lactam antibiotics against methicillin-sensitive and methicillin-resistant *S. aureus* [103]. In fact, when ampicillin was administered with dieckol (16 µg/mL), the antibiotic’s MIC against two conventional MRSA strains dropped dramatically from 512 to 0.5 µg/mL. MRSA resulted in being quite resistant to eckol derived from an ethyl acetate extract of the brown alga *E. cava* (MIC ranged from 125 to 259 µg/mL). However, in conjunction with ampicillin, the fractional inhibitory concentration (FIC) index of eckol shifted from 0.3 to 0.5 µg/mL, indicating a positive drug synergism against *S. aureus* [104].

PTs can modulate bacterial infections by downregulating antioxidant enzymes [45]. However, clinical studies and in vivo experiments are needed to delineate the mechanisms underlying the antibacterial activity of PTs as limited reports are available in this context, especially of those considering their potential adjuvant effect in conjunction with commercial drugs.

## 8. Anti-Biofilm and Antifouling Effects of PTs

Antibiotic abuse has been linked to antibiotic resistance in dangerous microorganisms, posing a global health risk [123]. Bacteria have the ability to attach themselves to solid surfaces and create biofilms, which are structured communities of bacteria [124]. Food can become an ideal growth medium for microbes, making it an easy target for the formation of biofilms. Bacterial drug resistance is a growing concern for modern medicine along with the induction of recurrent infections due to bacterial biofilm production [96]. Biofilms are linked to 65–80% of all bacterial illnesses, making them a difficult-to-solve healthcare issue. Biofilms are microbial communities that are wrapped in a complex polymeric (polysaccharide) structure called glycocalyx. In humans and animals, such biofilms can escape innate and adaptive immune mechanisms [105,125]. They are characterized by a higher incidence of horizontal DNA transfer, which leads to antibiotic and multidrug resistance. Reproduction foci arise periodically in some zones of the biofilm, from which free (planktonic) microorganisms are released into the environment. Microorganisms within a biofilm are more protected from harsh environmental conditions, antimicrobial medicines, and the immunological defenses of the host organism [106,126,127,128]. Inflammatory illnesses of the oral cavity are associated with the production of bacterial biofilms [129,130].

Algae have a number of mechanisms for preventing the aggregation and colonization of undesired organisms, such as pathogenic microbes. Diterpenoids, volatile compounds, fucoidans, PTs, fucoxanthins, and other chemicals found in algae have antimicrobial activity against bacteria, fungi, and viruses. Brown algae, such as *Fucus*, *Bifurcaria*, *Cystoseira*, and *Sargassum*, contain polyphenols that have antibacterial properties [131,132]. Antibacterial and antifouling properties have been also documented in green algae, such as *Ulva*, thanks to the presence of chlorophylls and *β*-carotene [133]. With reference to PTs, the antibiofilm properties can be attributed to their characteristic of being polyphenolic compounds. Enzymatic extraction of polyphenols from brown alga *Sargassum muticum’s* provided evidence of their antibiofilm capacity [66].

In the last few years, foodborne illnesses have been a major public health concern [134]. Food contaminated with *E. coli*, which generates the Shiga toxin, caused serious epidemics as a foodborne disease [101,135]. *E. coli* serotypes O113:H21 and O154:H10 developed biofilms on surfaces of food contact points [99,136]. The antibacterial and antibiofilm activities of PTs from the brown alga *Ascophyllum nodosum* have been evaluated against two Shiga toxin-producing *E. coli* strains (serotypes O113:H21 and O154:H10) resulting in the inhibition of the formation of the biofilms of both strains within 24 h of incubation [121]. Both strains overcame the inhibitory effect of PTs after 72 h, and the biofilm parameters reached control levels (6.4 and 6.2 log_10_ CFU/cm^2^ in the PTs sample and control, respectively). PTs inhibited cell growth and synthesis of exopolysaccharides in *E. coli* [121].

The anti-biofilm action of PTs are also due to their anti-adhesive properties and quorum sensing suppression (QS) [95]. The phenolic compounds in the methanol extracts of the brown algae *Halidrys siliquosa* were subsequently extracted by hexane/ethyl acetate (1:1 ratio) and evaluated against *S. aureus* and found to be sensitive, with MIC and minimum bactericidal concentration (MBC) values ranging from 0.1562 to 0.3125 mg/mL [105]. Minimum biofilm eradication concentration values of 1.25 mg/mL and 5 mg/mL indicated that biofilms of *S. aureus* MRSA 33593 and *S. aureus* MRSA 10442 are also sensitive to the mixture. Algal PTs-rich extract from *A. nodosum* was used for destroying biofilms by modulating the oxidative stress against biofilm-forming bacteria, *Porphyromonas gingivalis* and *Streptococcus gordonii,* which cause inflammatory disorders of the oral cavity. The Ag-zeolite-PT complex showed a strong bactericidal effect on *P. gingivalis* but was ineffective against *S. gordonii,* although it could hinder its biofilm development. The phenolic extract had no bactericidal or antibiofilm activity, still significantly reduced the secretion of inflammatory cytokines, such as tumor necrosis factor alpha (TNFα) and interleukin 6 (IL-6) in LPs-stimulated macrophages, along with lowered lipid peroxidation in gingival epithelial cells [106].

## 9. Conclusions and Future Perspectives

Marine brown algae hold a crucial position as an almost inexhaustible source of biologically active chemicals with a broad spectrum of medicinal value. PTs have a lot of potential for therapeutic applications, such as antioxidant, antiviral, antithrombotic, fungicidal, neuroprotective, and anticancer agents. As reported in the literature, brown algae derived PTs have antibacterial capabilities that are particularly appealing to be used against a wide spectrum of pathogenic microbes, including MRSA, while being non-toxic to healthy cells. Standardization of the procedures and conditions of extraction, as well as any subsequent treatment that might influence the degree of polymerization of the extract, are critical in accessing the PTs and their antibacterial activity. In addition, the extraction procedures must be highly reproducible in order to obtain reliable biological properties and they must rely on “environmentally-sustainable chemistry” principles.

The biological features depend on the structure of the final products and the correlation between them is currently being investigated to understand the antibacterial mode of action of these polyphenolic compounds. Numerous opinions on the mechanisms of action of various algal-derived bioactive compounds with antibacterial characteristics are available; but in context of PTs, it is still in its infancy. The interaction of PTs with the bacterial cell wall of both gram-positive and gram-negative bacteria, as well as changes in bacteria’s ultrastructural organization, has been critically viewed as the potential mechanisms; however, an exact dimension of mechanistic involvement has not been discussed. Possibly, PTs can also function by bacterial cell wall synthesis inhibition, protein synthesis inhibition, nucleic acid synthesis inhibition, oxidative imbalance and subsequent induction of cell death. Despite the many unanswered questions, such as unclear mode of action and lack of mechanistic inner view, the antimicrobial effect of PTs represent a viable starting point for developing new antimicrobial medications for the treatment and prevention of infectious diseases. Modern analytical techniques open up a slew of possibilities for extracting and investigating the structural diversity of these intriguing compounds, hopefully leading to the final repurposing their most active components as novel pharmaceuticals or prototypes for the development of new antibacterial drugs.

## Figures and Tables

**Table 1:** Summary of the in vitro antibacterial activity of phlorotannins.

Phlorotannins	Extract	Bacteria	Effect	Ref.
PTs aqueous extract	*Ericaria crinita* (formerly known as *Cystoseira crinita*)	*Klebsiella*, *Bacillus cereus*	MIC of 25 mg/mL MIC of 25 mg/mL	[95]
PTs ethyl acetate extract	*Ecklonia stolonifera* and *Ecklonia cava*	methicillin-resistant *Staphylococcus aureus* (MRSA)	antibacterial efficacy	[70]
Phlorofucofuroeckol-A	*E. bicyclics*	MRSA	inhibited bacterial growth	[38,98,99]
Low molecular weight PTs	*Sargassum thunbergia*	*Vibrio parahaemolyticus*	cell membrane and cell wall damage, facilitating cytoplasm leakage and membrane permeability	[100]
Phlorofucofuroeckol derivative	*E. bicyclics*	*Propionibacterium*	MIC of 32 g/mL; reduced resistance to erythromycin and lincomycin	[101]
Phlorofucofuroeckol	*Eisenia bicyclis*	MRSA	inhibited expression of mecI, mecR1, and mecA genes and regulated expression of methicillin resistance by suppressing penicillin-binding protein 2a production	[38,102]
Dieckol	*E. stolonifera*	MRSA	synergistic effect with ampicillin (MIC from 512 to 0.5 mg/mL)	[103]
Eckol	*E. cava*	*S. aureus*	synergistic effect with ampicillin (eckol FIC from 0.3 to 0.5 µg/mL)	[104]
PTs extract	*Ascophyllum nodosum*	*E. coli*	inhibition of biofilm formation within 24 h of incubation	[95]
PTs methanol extract	*Halidrys siliquosa*	*S. aureus*	MIC and MBC from 0.1562 to 0.3125 mg/mL	[105]
PTs-rich extract	*A. nodosum*	*Porphyromonas gingivalis*	significantly reduced secretion of inflammatory cytokines and lowered lipid peroxidation	[106]
